# Polymyxin B-Induced Kidney Injury Assessment of a Novel Formulation of Polymyxin B (VRP-034) in Rats

**DOI:** 10.3390/antibiotics10040359

**Published:** 2021-03-28

**Authors:** Dilip Roy, Amol Kulkarni, Manu Chaudhary, Saransh Chaudhary, Anurag Payasi, Anmol Aggarwal

**Affiliations:** Venus Medicine Research Centre, Baddi 173205, Himachal Pradesh, India; vmrc_hod3@venusremedies.com (D.R.); vmrc_staff2@venusremedies.com (A.K.); research@venusremedies.com (M.C.); saransh@venusremedies.com (S.C.); anmolaggarwal@venusremedies.com (A.A.)

**Keywords:** drug-induced kidney injury, polymyxin B, acute kidney injury, KIM-1, nephrotoxicity, cystatin-C, biomarkers

## Abstract

Despite the crucial role of Polymyxin-B in treating life-threatening gram-negative infections, its clinical utility is limited due to the risk of acute kidney injury. In response, a novel formulation of polymyxin-B is being developed to mitigate drug-induced kidney injury. In this study, we have assessed the toxicity of four variants of that novel formulation (VRP034_F21-F24) in comparison with standard polymyxin-B using kidney injury biomarkers in rats. Sprague-Dawley rats were subcutaneously administered either polymyxin-B (control) or one of the four polymyxin-B formulations at a dose of 25 mg/kg/day (HED: 4 mg/kg/day) in four divided doses for two days. Serum samples were collected at baseline and at the end of day 2 for the determination of serum biomarkers. Necropsy was done on day 2 and kidney was collected for histopathological evaluation. In the control group, statistically significant increase (*p* < 0.0001) in all biomarkers was observed on day 2 as compared to baseline values [urea: 311%; creatinine: 700%; KIM-1: 180%; cystatin-C: 66%] and 50% of the animals died (one after the 7th dose and two after the 8th dose) before scheduled necropsy. In contrast, animals treated with novel formulations did not show a significant increase across any of the biomarkers and no mortality was observed. Histopathology of the control group kidney confirmed necrotic changes in tissues with congestion and vacuolization, whereas only minor tubular damage was noted in two formulation groups (VRP034_F21, F24) and no appreciable damage was detected in the other two groups (VRP034_F22-23). The novel formulation of polymyxin-B tested in this study significantly reduced the risk of polymyxin-induced kidney injury in rats.

## 1. Introduction

The threat of increasing bacterial resistance to antibiotics has been a global concern since the last decade. It is directly associated with a substantial increase in morbidity, mortality, and treatment costs [1]. The situation is further complicated due to the absence of new antibiotics, and has forced the medical community to resort to decades-old toxic drugs like polymyxins. Although this group of polypeptide antibiotics consists of five chemically different compounds (polymyxins A–E), only two forms are systemically active: polymyxin B and polymyxin E (colistin) [2]. The use of polymyxins has been associated with compromised patient safety due to its ability to induce kidney injury. Polymyxin-induced kidney injury is a major stumbling block in the effective clinical use of these drugs against multidrug resistant (MDR) bacterial infections. The exact mechanism by which polymyxin B imparts toxicity in the kidney is yet to be fully elucidated; however, in vivo studies performed in rats suggested that it arises due to its extensive reabsorption and subsequent accumulation in the renal proximal tubule. This accumulation is reported to be mediated through endocytic receptors, megalin, and other transporters present in the renal proximal tubule [3]. Megalin comprises low-density lipoproteins and members of the receptor protein family group which is extensively present in the microvilli of renal tubular cells. Polymyxin B is a polybasic/cationic drug with a high-binding affinity for these receptors. This receptor-mediated uptake leads to the accumulation of polymyxin B in the proximal tubule and results in cell death and kidney injury [4].

### 1.1. About VRP-034

Considering the increasing use of polymyxins in the treatment of MDR gram-negative infections and their dose-limiting toxicity, there is an urgent need for newer, better treatments. However, the current market dynamics for novel antibiotics and the antibiotic pipeline are not suggestive of an imminent solution [5]. Acknowledging this unmet medical need, Supra Molecular Cationic (SMC) complexes of polymyxin B (VRP-034_F21 to F24) were developed with the aim of reducing drug-induced kidney toxicity. These SMC complexes are designed to impair drug uptake and accumulation inside kidney cells. As per patent hypothesis, the SMC complex is engineered to cater to an orchestra of cellular mechanisms, including competitively inhibiting drug attachment to the receptor site (megalin, responsible for renal toxicity) via high-affinity like-charge binding, reducing oxidative stress through a cascade of reactions, improving the microcirculatory function essential for renal ROS and RNS homeostasis, and ultimately reduce the risk of kidney injury. 

The technology is based on electrostatic interactions to maintain drug properties without altering efficacy. The formulations (VRP-034_F21-F24) do not interfere with the efficacy of polymyxin B to kill gram-negative bacteria. We tested the in vitro efficacy of all the formulations against quality control and clinical isolates of *E. coli* [NCTC 13,353 (CTX-M); NCTC 13,476 (IMP)], *K. pneumoniae* [ATCC 1705 (KPC); NCTC 13,439 (VIM-1)], *P. aeruginosa* [clinical isolates (IMP- and VIM-positive, respectively)], and *A. baumannii* [NCTC13302 (OXA-25); clinical isolate (IMP-positive)] and found that all the minimum inhibitory concentrations (MICs) were within the acceptable range of dilutions as compared to standard polymyxin B (see Appendix A Table A1).

### 1.2. Kidney Injury Biomarkers

In most clinical settings, the extent of acute kidney injury (AKI) is quantified by biomarkers such as blood urea nitrogen (BUN) and creatinine (Cr). However, these biomarkers can only report kidney damage and cannot predict it. It is estimated that before creatinine is elevated to a clinically significant level, up to 50% of nephron function could be lost [6,7]. Therefore, there is a need for more sensitive and reliable biomarkers to detect AKI early during therapy. Two novel sensitive biomarkers, namely-Kidney Injury Molecule-1 (KIM-1) and cystatin C, provide means of early detection of AKI. KIM-1 concentration is known to get upregulated in proximal tubular cells after kidney injury, and hence, it serves as a biomarker for kidney impairment. A positive correlation is established between the increasing period of ischemia and blood levels of KIM-1 [8]. Further, its presence in both rodents and humans supports its use as a specific biomarker for AKI; therefore, its application has been permitted by both the US Food and Drug Administration and European Medicines Agency for the detection of drug-induced kidney injury in rats as well as in clinical studies [9,10,11,12]. Cystatin C is a protein marker that is freely filtered at the glomerulus and then reabsorbed by the renal tubular epithelium. The clinical use of cystatin C to evaluate renal function has been increasing recently. Cystatin C is considered a specific marker for glomerular filtration rate (GFR) assessment. Many independent studies have demonstrated that a combination of serum cystatin C with serum creatinine may more accurately detect minor alterations in GFR than either marker alone [11,12,13].

The present study evaluated four different variants of the novel formulation of polymyxin B (VRP034_F21–24) in comparison with standard polymyxin B with regard to their ability to reduce the polymyxin B-induced kidney injury in rats using both traditional (urea and creatinine) and novel biomarkers (KIM-1 and cystatin C), and histopathological evaluation.

## 2. Results

### 2.1. Kidney Injury Biomarkers Examination

**Figure 1:**
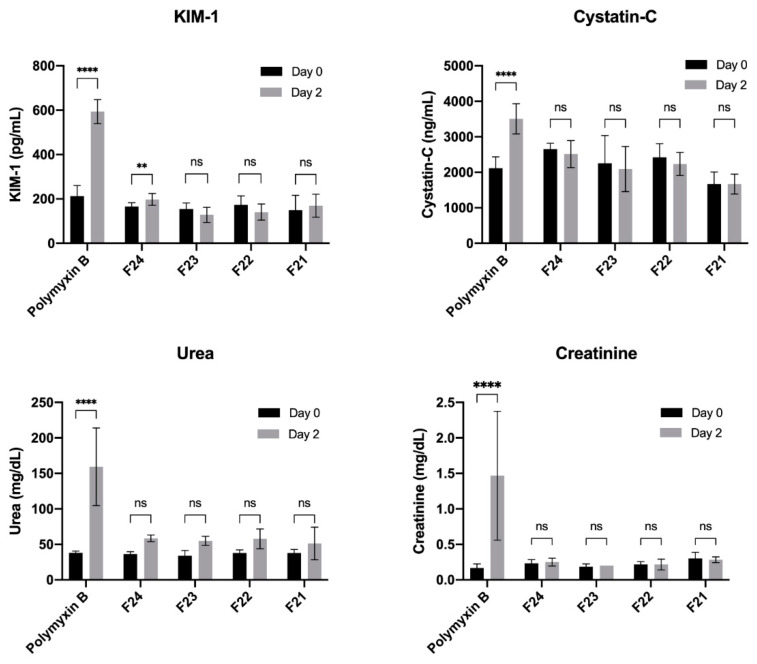
Biomarker response in rats before (day 0) and after treatment (day 2) with novel formulations VRP034_F21–24 and polymyxin B (control). Note: (i) In the polymyxin B (control) group, one animal died after the 7th dose and the other two died after the 8th dose. The results shown are for animals who survived treatment; *n* = 3. (ii) In all other treatment groups, no mortality was observed, and the results are shown for all animals; *n* = 6. (iii) *p*-values are presented for increase in biomarkers on day 2 vs. day 0; nonsignificant (ns); *p* < 0.01 (**); *p* < 0.0001 (****).

The traditional and novel biomarker values of all the groups are summarized in Table 1 and graphically displayed in Figure 1. The baseline (day 0) values indicate concentrations of biomarkers in the serum before treatment, and the day 2 values indicate the concentrations after the eighth dose, before scheduled necropsy. In the control group, all the biomarkers showed a significant increase in their values on day 2 as compared to the baseline. Urea and creatinine levels increased by 311% and 700% respectively, and KIM-1 and cystatin-C levels increased by 180% and 66% respectively. The increase in all the four biomarkers in the control group was found to be extremely statistically significant (*p* < 0.0001). On the other hand, all the SMC complex formulations of polymyxin B mitigated these changes and showed better results. Animals treated with these formulations did not show any significant increase across all four biomarkers on day 2 (*p* > 0.05), except for KIM-1 in formulation VRP034_F24 that increased by 19% (*p* < 0.01). Also, animals treated with VRP034_F22 and VRP034_F23 showed decreased KIM-1 (19% and 17%) and cystatin C (8% and 7%) levels; however, these levels were within the normal range of KIM-1 and cystatin-C expression in rats. The biomarker expression levels observed after treatment with standard polymyxin B (control) and VRP-034 formulations were significantly different.

### 2.2. Clinical Signs Observations

The clinical manifestations across all treatment groups are highlighted in Table 2. Animals treated with control drug showed immediate clinical manifestations like pallor of the ears and pads with redness, muscular incoordination, and respiratory distress which persisted for 2–3 h. On the second day, these animals showed complete flaccidity of skeletal muscle with dyspnea interrupted by gasping respiration after 30 min of dosing which persisted for 2–3 h. As a result, three animals (out of six) died in the control group (one animal died after the seventh dose and the other two after the eighth dose) before scheduled necropsy. However, none of the animals dosed with the novel formulations (VRP-034_F21-F24) showed any signs of severe toxicity. Only a few animals in the VRP-034_F21, F24 groups exhibited muscle incoordination and redness of ear. Furthermore, none of the animals died in these groups.

### 2.3. Histopathological Evaluation

**Figure 2:**
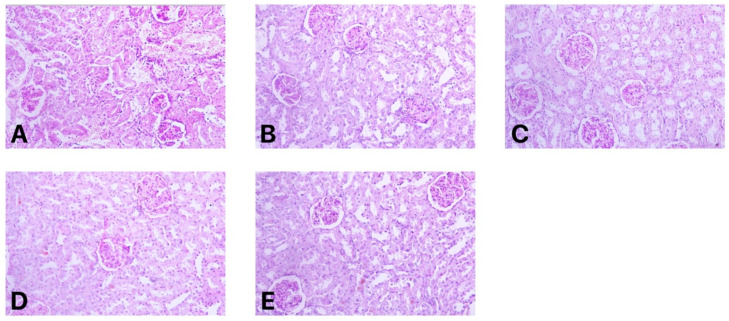
Histopathological images of rat kidneys; (**A**) Polymyxin B (Control)-Showing necrotic changes in kidney tissues with congestion and vacuolization. (**B**) VRP-034_F21-Kidney showing normal organization of glomeruli, mild tubular damage with vacuolization. (**C**) VRP-034_F22- Showing normal glomerular and tubular histology. The tubules were largely intact without the presence of any mononuclear infiltrates in the interstitium, and blood vessels were also unremarkable. (**D**) VRP-034_F23-Showing normal glomerular and tubular histology. The tubules were largely intact without the presence of any mononuclear infiltrates in the interstitium and blood vessels were also unremarkable. (**E**) VRP-034_F24-Showing normal organization of glomeruli, mild tubular damage with vacuolization.

One random kidney from each group was collected for histopathological evaluation and the images are displayed in Figure 2. Histopathologic examination of kidneys in rats treated with control drug revealed necrotic changes in the kidney tissues with congestion and vacuolization. VRP034_F21 and F24 treated kidneys showed normal organization of glomeruli but mild tubular damage with vacuolization. These microscopic findings are indicative of drug-induced kidney injury. On the other hand, VRP034_F22 and F23 treated kidneys showed normal glomerular and tubular histology. The tubules were largely intact without the presence of any mononuclear infiltrates in the interstitium, and blood vessels were also unremarkable. These variants did not show any interstitial nephritis or tubular damage. 

## 3. Discussion

Polymyxins are considered as the last resort antibiotic for the treatment of life-threatening gram-negative infections. However, the toxicity associated with this class of drugs has limited its clinical use. Its use is further restricted due to inadequate understanding of PK/PD. However, limited PK/PD studies have indicated that with the currently approved dosing regimens, polymyxins achieve suboptimal concentrations in plasma in many patients, and increasing the plasma concentrations by increasing the dose is associated with increased risk of AKI [14]. Consequently, the utility of polymyxin B can be improved by selectively reducing its nephrotoxicity. It has been observed that even therapeutic doses of polymyxin B are associated with toxicity, and that the major reason for this toxicity is the accumulation of the drug in the kidney proximal tubule. This toxicity can be quantitatively measured using traditional (serum urea and serum creatinine) and novel kidney biomarkers (KIM-1 and cystatin-C) [8,9,10,11,12,13]. Usually, traditional biomarkers are best for reporting kidney injury, whereas novel biomarkers are more sensitive, and their early expression is helpful in predicting kidney injury. Many efforts had been made to reduce the toxicity of polymyxins, but a viable solution is yet to be found.

Studies of polymyxin B in rats have clearly exemplified extensive reabsorption followed by accumulation of polymyxin B in the proximal tubule [4]. Our study also corroborates this observation. The group treated with polymyxin B (control) showed a significant elevation in both traditional and novel biomarkers. This elevation is a reflection of kidney damage and was verified by histopathology. Rat kidneys treated with polymyxin B showed observable necrotic changes in kidney tissues with congestion and vacuolization (Figure 2). This observation is common under clinical conditions as well, and polymyxin B is consistently reported to have a toxic effect at approved doses. This dose–toxicity relationship for polymyxin B is critical, as approved doses give suboptimal efficacy and are also associated with toxicity. Therefore, in order to achieve optimal efficacy, it is necessary to reduce polymyxin B-associated toxicity. 

With the aim of solving this problem, Supra Molecular Cationic (SMC) complexes of polymyxin B (VRP-034_F21 to F24) have been developed. This technology aims to reduce kidney toxicity associated with polybasic/cationic drugs without reducing efficacy. Four variants of VRP-034 (F21–F24) were tested. Polymyxin B dose of 25 mg/kg/day (HED: 4 mg/kg/day) was administered in four divided doses for two days. The 4 mg/kg/day dose is 33% higher than highest approved human dose of polymyxin B. While the higher dose is ideal to augment drug exposure and improve the odds of microbiological eradication, it is associated with an increased risk of AKI. In this study, we assessed the performance of SMC complexes vs. regular polymyxin B in inducing kidney injury in rats at the high dose.

All of the novel formulations were found to be safer concerning their toxic effect on kidney compared to the marketed formulation of polymyxin B (control). No significant elevation in baseline values across all biomarkers after treatment indicated that the novel formulations reduce toxicity associated with polymyxin B. This statement is supported by the histopathological result as well, which clearly demonstrated that treatment with novel formulations did not result in tubular damage, unlike polymyxin B. The absence of such tubular damage is directly related to the absence of accumulation of polymyxin B.

In order to evaluate AKI, more sensitive biomarkers are needed. Novel biomarkers are important in the detection of AKI in the early stages of therapy. KIM-1 and cystatin-C fulfil this requirement, and were found to be more consistent, sensitive and provided convincing values with which to differentiate the extent of nephrotoxicity of all the tested formulations. Also, the importance of histopathological evaluation is greater in such studies. Such an evaluation is supportive of biomarker data and confirms the result. In the current study, kidney sections of the treated rats efficiently depicted and differentiated the extent of kidney damage caused by polymyxin B (control) and lack thereof in the novel SMC complex formulations.

However, there are limitations to our study. Due to the high dose of polymyxin B tested in the study, we could not evaluate the long-term safety of the SMC complexes over chronic administration (6–7 days). Considering the mechanism of action of the SMC complexes involve competitive inhibition, it is important to evaluate the safety profile in a long-term study, albeit at a lower daily dose to reduce neurological distress. This shall constitute part of our future work.

In conclusion, the results highlight that all the variants of VRP-034 formulation significantly mitigated the risk of polymyxin B-induced kidney injury. Also, VRP034_F22–23 outperformed other formulations across biomarkers, clinical manifestations, and histopathological examination. Further in vivo studies on VRP-034 will help to fully elucidate its role in reducing polymyxin-induced kidney injury and to establish its clinical utility in treating resistant gram-negative infections.

## 4. Materials and Methods

### 4.1. Compounds

The SMC complexes of Polymyxin B-VRP034_F21, VRP034_F22, VRP034_F23 and VRP034_F24 were developed at Venus Medicine Research Centre, India. The SMC complexes were prepared by complexing the cationic drug (polymyxin B) with like-charge molecules by altering their property from basic to acidic, without chemical cross linking. The complexes comprise of (a) polymyxin B sulphate (b) a cationic compound selected from the group of amino acids like l-lysine, l-arginine, l-histidine; (c) a macromolecule base selected from a group of natural polysaccharides (like low molecular weight dextran, chitosan, heparin, dextran sulphate) as scaffold without chemical modification. The complexes are formed by cationic electrostatic interactions in specified charge molecular weight relationship. The ratio of polymyxin B: cationic amino acid: scaffold base in all the novel formulations (VRP034_F21–F24) lies between 1:0.1:0.1 to 1:3:1. The complex such formed is then lyophilized. All the novel formulations and the marketed polymyxin B had 50 mg (or 500,000 IU) of polymyxin B base activity before dilution. Polymyxin B sulphate was purchased from Xellia Pharmaceuticals, Denmark. Standard polymyxin B used in the experiment as control was manufactured by Bharat Serum and Vaccines, Mumbai, India. The stock solutions of all the drugs were reconstituted in distilled water.

### 4.2. Animals

The study was conducted on healthy female Sprague Dawley (SD) rats of 8–10 weeks of age, weighing in the range of 275–375 g. They were procured from National Institute of Pharmaceutical Education and Research (NIPER), Mohali, Punjab, India. Animals were handled in accordance with the ethical principles and standards laid out in the Committee for the Purpose of Control and Supervision of Experiments on Animals (CPCSEA) guidelines. Animals were kept under 12 h light and 12 h dark cycles and during experiment supplied with food and water ad libitum. The study was approved by the Animal Ethics Committee.

### 4.3. Study Design

In total, 30 animals were used in the study, divided into five groups, with six animals in each group. Groups were treated either with standard polymyxin B or one of the four novel formulations of polymyxin B (VRP034_F21–24) at a total daily dose equivalent to 25 mg/kg/day (6.25 mg/kg q6 h) of polymyxin B for two days via subcutaneous administration. The dose allometrically scales to a dose of 4 mg/kg/day in humans [15]. The serum samples were collected at baseline (day 0) and at the end of day 2 and kept frozen at −80 °C for analysis of four biomarkers, KIM-1, cystatin-C, urea and creatinine. The levels of biomarker were calculated using ELISA kits. Quantikine ELISA, KIM-1 and cystatin-C kits were procured from R&D systems (Minneapolis, MN, USA), urea and creatinine kits were procured from BioSystems (Barcelona, Spain). 

Animals were observed daily for clinical signs and mortality. On day 2, after collection of serum samples, animals were euthanized, and kidneys were removed through necropsy for gross observation and histopathological evaluation. The change in serum biomarkers from baseline across all formulations was statistically analyzed and biomarker results were cross validated using histopathological assessment.

### 4.4. Blood Sampling

Blood samples were collected on baseline and day 2 after treatment through the retro-orbital route. After withdrawal, the blood samples were allowed to clot for about half an hour. These samples were then centrifuged at 3000 rpm for 15 min. The supernatant collected was then stored at −80 °C until further analysis. 

### 4.5. Quantification of Serum Biomarkers of Kidney Injury

Concentrations of traditional biomarkers, i.e., creatinine and urea, were analyzed using a biochemistry analyzer (Biosystems A15), whereas for novel biomarkers, KIM-1 and cystatin-C, concentrations were detected using ELISA kits.

### 4.6. Statistical Analysis

The results were analyzed and expressed as mean ± SD. The statistical comparison for biomarker values was made between day 2 and baseline (day 0) values. The data were evaluated using repeated measures two-way ANOVA followed by Bonferroni’s post hoc test. Data were adjusted for multiple comparisons and the multiplicity adjusted *p*-values were reported. The complete analysis was performed using GraphPad Prism (V9.0; GraphPad Software, San Diego, CA, USA).

### 4.7. Histopathological Evaluation

After completion of treatment and collection of serum samples, animals were humanely euthanized by carbon dioxide asphyxiation. Kidneys were isolated, washed in normal saline and preserved in 10% neutral buffer formalin (NBF) for tissue fixation for 48 h. Formalin-fixed kidneys were trimmed both longitudinally and horizontally and processed in an automatic tissue processor (Medimeas) for 12 h. Block was prepared by embedding processed tissue in paraffin wax. It was then sliced at 5 microns with the help of microtome (Medimeas) and slides were prepared. Slides were stained with periodic acid and Schiff’s reagent (PAS staining). After staining, these slides were observed under a microscope (Nikon Eclipse 80i, 20X magnifying lens, Tokyo, Japan). Photomicrographs were taken using the camera (Nikon DS-Ri1, Tokyo, Japan) attached to the microscope. Slides were observed thoroughly for any lesions in the glomerular and tubular parenchyma. 

## Figures and Tables

**Table 1:** Mean Biomarker values at Baseline (day 0) and day 2 of novel formulations (VRP034_F21–24) and Polymyxin B (Control) ^1,2,3,4^.

Drug	Day 0	Day 2	Elevation in Values	*p*-Value
**KIM-1 (pg/mL)**				
Polymyxin B	212.32 ± 47.86	593.56 ± 54.18	180%	<0.0001
VRP-034_F21	149.33 ± 67.15	169.25 ± 51.51	13%	>0.05
VRP-034_F22	173.66 ± 39.80	140.43 ± 36.23	−19%	>0.05
VRP-034_F23	154.34 ± 27.22	128.23 ± 34.32	−17%	>0.05
VRP-034_F24	165.78 ± 16.99	197.39 ± 26.59	19%	<0.01
**Cystatin C (ng/mL)**				
Polymyxin B	2113.11 ± 323.86	3506.17 ± 425.68	66%	<0.0001
VRP-034_F21	1666.69 ± 341.52	1668.2 ± 279.12	0.1%	>0.05
VRP-034_F22	2424.05 ± 382.87	2235.28 ± 324.24	−8%	>0.05
VRP-034_F23	2252.62 ± 783.90	2092 ± 635.51	−7%	>0.05
VRP-034_F24	2654.05 ± 164.94	2512.53 ± 382.49	−5%	>0.05
**Urea (mg/dL)**				
Polymyxin B	38.77 ± 2.45	159.33 ± 54.63	311%	<0.0001
VRP-034_F21	37.80 ± 5.05	51.25 ± 23.02	36%	>0.05
VRP-034_F22	37.88 ± 4.33	57.77 ± 13.93	52%	>0.05
VRP-034_F23	34.1 ± 7.34	54.98 ± 6.43	61%	>0.05
VRP-034_F24	36.3 ± 3.46	58.5 ± 4.65	61%	>0.05
**Creatinine (mg/dL)**				
Polymyxin B	0.19 ± 0.04	1.48 ± 0.09	700%	<0.0001
VRP-034_F21	0.31 ± 0.08	0.26 ± 0.04	−16%	>0.05
VRP-034_F22	0.21 ± 0.03	0.22 ± 0.07	5%	>0.05
VRP-034_F23	0.20 ± 0.04	0.19 ± 0.02	−3%	>0.05
VRP-034_F24	0.23 ± 0.02	0.25 ± 0.02	8%	>0.05

^1^ Values are mean ± SD (*n* = 6). ^2^ Polymyxin B refers to the standard formulation currently available in the market. ^3^ Formulations VRP-034_F21–24 represents novel formulations of polymyxin B. ^4^ *p* values are presented for increase in biomarkers on day 2 vs. day 0.

**Table 2:** Clinical observations in the polymyxin B (control) and novel formulation groups (VRP034_F21–24).

	Polymyxin B	VRP-034_F21	VRP-034_F22	VRP-034_F23	VRP-034_F24
Flushing/Blushing (redness of pads)	Severe	Moderate	Mild	Mild	Moderate
Abnormal gait (Muscular Incoordination)	Severe	Moderate	Mild	Mild	Moderate
Akinesia	Moderate	Mild	Mild	Mild	Mild
Dyspnoea (Breathlessness)	Severe	Mild	Mild	Mild	Mild
Flaccidity (Grip strength)	Severe	Moderate	Mild	Mild	Moderate
Morbidity	Severe	Mild	Mild	Mild	Mild

## Data Availability

The data presented in this study are available on request from the corresponding author. The data are not publicly available due to intellectual property consideration.

## Appendix A

**Table A1:** Competitive MICs of all the Polymyxin B formulations against gram-negative quality control and clinical isolates.

Microorganisms	Polymyxin B	VRP-034_F21	VRP-034_F22	VRP-034_F23	VRP-034_F24
	**MIC (µg/mL)**				
*E. coli* NCTC 13353 (CTX-M)	0.0625	0.0625	0.0312	0.125	0.0625
*E. coli* NCTC 13476 (IMP)	0.125	0.125	0.0625	0.25	0.125
*K. pneumoniae* ATCC 1705 (KPC)	0.25	0.5	0.125	0.125	0.25
*K. pneumoniae* NCTC 13439 (VIM-1)	0.125	0.25	0.0625	0.0625	0.125
*A. baumannii* NCTC13302 (OXA-25)	0.125	0.25	0.125	0.0625	0.0312
*A. baumannii* Clinical isolate (IMP positive)	0.125	0.25	0.0625	0.125	0.125
*P. aeruginosa* Clinical isolate (IMP positive)	0.25	0.125	0.25	0.0625	0.125
*P. aeruginosa* Clinical isolate (VIM positive)	0.5	0.25	1.0	0.25	0.5

## References

[1] Akova M. (2016). Epidemiology of antimicrobial resistance in bloodstream infections. Virulence.

[2] Avedissian S.N., Liu J., Rhodes N.J., Lee A., Pais G.M., Hauser A.R., Scheetz M.H.A. (2019). Review of the clinical pharmacokinetics of polymyxin b. Antibiotics.

[3] Manchandani P., Zhou J., Babic J., Ledesma K., Truong L., Tam V. (2017). The role of renal drug exposure in polymyxin B-induced nephrotoxicity. Antimicrob. Agents Chemother..

[4] Zavascki A.P., Nation R.L. (2017). Nephrotoxicity of polymyxins: Is there any difference between colistimethate and polymyxin B?. Antimicrob. Agents Chemother..

[5] Beyer P., Paulin S. (2020). The antibacterial research and development pipeline needs urgent solutions. ACS Infect. Dis..

[6] Bellomo R., Ronco C., Kellum J.A., Mehta R.L., Palevsky P. (2004). Acute dialysis quality initiative workgroup. Acute renal failure-definition, outcome measures, animal models, fluid therapy and information technology needs: The Second International Consensus Conference of the Acute Dialysis Quality Initiative (ADQI) Group. Crit. Care.

[7] Waikar S.S., Betensky R.A., Emerson S.C., Bonventre J.V. (2013). Imperfect gold standards for biomarker evaluation. Clin. Trials.

[8] Bonventre J.V., Vaidya V.S., Schmouder R., Feig P., Dieterle F. (2010). Next-generation biomarkers for detecting kidney toxicity. Nat. Biotechnol..

[9] Sabbisetti V.S., Waikar S.S., Antoine D.J., Smiles A., Wang C., Ravisankar A., Ito K., Sharma S., Ramadesikan S., Lee M. (2014). Blood kidney injury molecule-1 is a biomarker of acute and chronic kidney injury and predicts progression to ESRD in type I diabetes. J. Am. Soc. Nephrol..

[10] United States Food and Drug Administration List of Qualified Biomarkers. https://www.fda.gov/drugs/cder-biomarker-qualification-program/list-qualified-biomarkers.

[11] Dieterle F., Sistare F., Goodsaid F., Papaluca M., Ozer J.S., Webb C.P., Baer W., Senagore A., Schipper M.J., Vonderscher J. (2010). Renal biomarker qualification submission: A dialog between the FDA-EMEA and predictive safety testing consortium. Nat. Biotechnol..

[12] Li Z., Shen C., Wang Y., Wang W., Zhao Q., Liu Z., Wang Y., Zhao C. (2016). Circulating kidney injury molecule-1 is a novel diagnostic biomarker for renal dysfunction during long-term adefovir therapy in chronic hepatitis B. Medicine.

[13] Inker L.A., Schmid C.H., Tighiouart H., Eckfeldt J.H., Feldman H.I., Greene T., Kusek J.W., Manzi J., Van Lente F., Zhang Y.L. (2012). Estimating glomerular filtration rate from serum creatinine and cystatin C. N. Engl. J. Med..

[14] Abdelraouf K., Braggs K.H., Yin T., Truong L.D., Hu M., Tam V.H. (2012). Characterization of polymyxin B-induced nephrotoxicity: Implications for dosing regimen design. Antimicrob. Agents Chemother..

[15] Nair A.B., Jacob S.A. (2016). Simple practice guide for dose conversion between animals and human. J. Basic Clin. Pharma..

